# Risk of Criminal Victimisation in Outpatients with Common Mental Health Disorders

**DOI:** 10.1371/journal.pone.0128508

**Published:** 2015-07-01

**Authors:** Sabine C. Meijwaard, Martijn Kikkert, Liselotte D. de Mooij, Nick M. Lommerse, Jaap Peen, Robert A. Schoevers, Rien Van, Wencke de Wildt, Claudi L. H. Bockting, Jack J. M. Dekker

**Affiliations:** 1 Arkin Institute for Mental Health Care, PO Box 75848, 1070 AV, Amsterdam, The Netherlands; 2 University of Groningen, University Medical Centre Groningen, Department of Psychiatry, Hanzeplein 1, 9713 GZ, Groningen, The Netherlands; 3 University of Groningen, Faculty of Behavioural and Social Sciences, Department of Clinical Psychology, Grote Kruisstraat 2–1, 9721 TS, Groningen, The Netherlands; 4 University Utrecht, Faculty of Behavioural and Social Sciences, Department of Clinical Psychology, Padualaan 14, 3584 CH, Utrecht, The Netherlands; 5 Free University of Amsterdam, Department of Clinical Psychology, Room 2B-73, Van der Boechorststraat 1, 1081 CD, Amsterdam, The Netherlands; Univ of Toledo, UNITED STATES

## Abstract

**Background:**

Crime victimisation is a serious problem in psychiatric patients. However, research has focused on patients with severe mental illness and few studies exist that address victimisation in other outpatient groups, such as patients with depression. Due to large differences in methodology of the studies that address crime victimisation, a comparison of prevalence between psychiatric diagnostic groups is hard to make. Objectives of this study were to determine and compare one-year prevalence of violent and non-violent criminal victimisation among outpatients from different diagnostic psychiatric groups and to examine prevalence differences with the general population.

**Method:**

Criminal victimisation prevalence was measured in 300 outpatients living in Amsterdam, The Netherlands. Face-to-face interviews were conducted with outpatients with depressive disorder (n = 102), substance use disorder (SUD, n = 106) and severe mental illness (SMI, n = 92) using a National Crime Victimisation Survey, and compared with a matched general population sample (n = 10865).

**Results:**

Of all outpatients, 61% reported experiencing some kind of victimisation over the past year; 33% reported violent victimisation (3.5 times more than the general population) and 36% reported property crimes (1.2 times more than the general population). Outpatients with depression (67%) and SUD (76%) were victimised more often than SMI outpatients (39%). Younger age and hostile behaviour were associated with violent victimisation, while being male and living alone were associated with non-violent victimisation. Moreover, SUD was associated with both violent and non-violent victimisation.

**Conclusion:**

Outpatients with depression, SUD, and SMI are at increased risk of victimisation compared to the general population. Furthermore, our results indicate that victimisation of violent and non-violent crimes is more common in outpatients with depression and SUD than in outpatients with SMI living independently in the community.

## Introduction

Psychiatric patients are considerably more likely to be the victim than the perpetrator of violent crimes [1–3]. Yet, scientific research on the relation between violence and mental illness has mainly focused on psychiatric patients as potential perpetrators. Psychiatric patients have a seriously increased risk of victimisation for both violent and non-violent crimes [1,4–13]. Prevalence rates are found to be 11 times higher in psychiatric patients than in the general population, even after controlling for demographic differences [14]. Criminal victimisation is a stressful event that impacts multiple domains of life, resulting in impaired occupational functioning, higher rates of unemployment and problematic intimate relationships [15]. Furthermore, victimisation in psychiatric patients causes increased use of psychiatric services, decreased quality of life, worsening of existing symptoms and elevated risk of revictimisation [15–17]. Victimisation in psychiatric patients is found to be associated with alcohol abuse or illicit drug abuse [1,7,9,18,19], psychotic symptoms [18], younger age, early onset of disorder and co-occurring disorders such as depression and personality disorder [5,9]. Additionally, it is found that victimisation correlates with the severity of symptoms [1,9,20].The prevalence of victimisation in psychiatric patients has been the topic of several studies in the past two decades. However, the majority of victimisation studies focused on inpatients or a mixed sample of in- and outpatients, mainly with a diagnosis of severe mental illness (SMI). In a systematic literature review performed by Maniglio [21] prevalence rates of violent victimisation in patients with SMI are estimated between 4% to 35%, whereas prevalence rates of non-violent crimes range from 8% to 28%. A cross-sectional prevalence study among 936 patients with SMI living in Chicago found that more than a quarter of the patients had been victim of a crime, a prevalence more than 11 times higher than the general population, even after controlling for demographic differences [14]. A recent Dutch victimisation study among outpatients with SMI [13] reported that 47% of the participants had fallen victim to a crime in the past year, with a relative risk for violent victimisation (sexual harassment, threats of violence and physical assault) 2.8 times higher than the general population. Also for patients with a substance use disorder (SUD) victimisation prevalence appears to be increased [22–25]. Prevalence of victimisation in patients with SUD ranges from 13% over the past 3 months [22] to 78% over the past year [23]. A study that examined intimate partner violence in outpatients with SUD found that 73% of the patients had fallen victim to psychological aggression, 51% to physical aggression and 33% reported injury following violent victimisation. Subgroups of SUD patients who appear to be especially vulnerable to victimisation are women, sex workers, homeless persons, recent perpetrators of violence and those with a history of poor mental health [23].

Moreover, several studies report that symptoms of depression are a risk factor for victimisation. However, most of these studies examine SMI patients with psychosis and a co-occurring depressive disorder. Little is known about the prevalence of victimisation in outpatients with depression. A study conducted in New Zealand [24] found that outpatients with a depressive disorder reported more victimisation in the form of physical assault than people without a mental disorder. However, this study is based solely on data from a 21-year-old birth cohort, so it is unclear if these results can be generalised to other age groups of depressive patients. Furthermore, Dienemann and colleagues [25] found that 61% of patients with depression had fallen victim to domestic violence and 30% reported to have experienced forced sex by their partner. This indicates that intimate partner violence is common in people who suffer from depression. A systematic review and meta-analysis on experiences of domestic violence and mental disorders revealed that women with depression have an increased risk of experiencing intimate partner violence (odds ratio = 2.77) [26]. Moreover, victimisation, particularly exposure to physical and sexual violence, is associated with an increased emergence of depression [27,28].

Due to methodological discrepancies, such as differences in operation and definitions that were used for ‘victimisation’ and ‘violence’, recall periods which vary from ten weeks [29] to three years [1], and differences in patient characteristics, it is difficult to compare victimisation prevalence between studies. Therefore, it remains unclear how victimisation prevalence of outpatients with depression compare to outpatients with SUD or SMI. Moreover, most studies do not compare the victimisation prevalence of psychiatric patients with a control sample from the general population.

### Aims of the study

The aims of this study are to determine 12-month violent and non-violent victimisation prevalence in outpatients with depressive disorder, SUD, SMI and the general population and to examine contextual differences regarding victimisation in these groups. Moreover, we analyse the predictors for both violent and non-violent victimisation.

## Materials and Methods

### Study design

This cross-sectional study was conducted among a sample of 300 psychiatric outpatients from mental health care institute Arkin or mental health care institute GGZinGeest, both situated in Amsterdam, The Netherlands. Trained psychology research associates carried out the surveys. The surveys were conducted face-to-face at the mental health care centre or at the home of the patient, according to the preference of the participant. Data was collected between January 17, 2011, and January 24, 2012.

### Population, inclusion and exclusion criteria

To be eligible for the study participants had to have a primary diagnosis of depressive disorder, including major depressive disorder, dysthymic disorder or depressive disorder not otherwise specified, according to The Diagnostic and Statistical Manual of Mental Disorders (DSM) [30], SUD or SMI. SUD was defined as a DSM-IV diagnosis of substance dependence or abuse of alcohol, amphetamines, cannabis, cocaine, opioids, hallucinogenic drugs and other forms of hard drugs. SMI was defined as a DSM-IV diagnosis of schizophrenia, schizoaffective disorder or bipolar disorder [30]. Additionally, SMI patients had to have received continuous intensive mental health care for more than two years. Furthermore, patients had to be over sixteen years old and had to have sufficient understanding of the Dutch language. All patients received outpatient care at Arkin or GGZinGeest, both public mental health care institutes, with catchment areas of treatment sites that together cover the entire Amsterdam region. Both institutes each treat approximately 25000 to 30000 patients annually with a broad spectrum of mental illnesses. Therapists at the outpatient clinics offered verbal and written information about this study to their patients. Subsequently, the therapist asked their patient if a research associate was allowed to contact them. Prior to the survey, patients received verbal and written information about the study. A written informed consent was obtained from all participants. Patients with a compromised ability to consent, as determined by their therapist, were not included in the study. Participants received a financial compensation of €15.

#### Patients with depressive disorder

During the time frame of the data collection, a total of 1100 patients with depressive disorder received outpatient treatment at PuntP, the specialised department for mood and anxiety disorders at Arkin Mental Health Care. In total 193 outpatients with depressive disorder were interested in participating in the study of which 58 eventually refused to participate or were not successfully contacted. Twenty-three patients did not meet the inclusion criteria for having sufficient understanding of the Dutch language. Furthermore, 10 patients were excluded because they did not have a primary diagnosis of depression or because the therapist indicated that participation in the study could have a negative effect on their treatment outcome. A total of 102 participants with a depressive disorder were included. None of the included patients had a current psychotic depression.

#### Patients with SUD

During the time frame of the data collection, a total of 293 patients with SUD received outpatient treatment at Jellinek, the specialised department for substance abuse treatment of Arkin Mental Health Care. A total of 106 participants expressed their interest in the study. All of these patients met the inclusion criteria and were all included. Of the included participants substance abuse was related to alcohol in 39 cases and to cannabis in 7 cases. Furthermore, 9 SUD patients were addicted to a form of hard drugs, and 50 were addicted to any combination of alcohol, cannabis and or hard drugs. One participant was dependent on benzodiazepines.

#### Patients with SMI

The victimisation survey in the SMI group was added to a follow-up assessment, that formed part of a longitudinal monitoring study for in- and outpatients with SMI in Amsterdam [31]. The inclusion criteria for this initial SMI study are described above. In 2005, 323 randomly selected SMI patients that received mental health care at Arkin or GGZinGeest were included for this study. Of the patients that were included in 2005, most (73%) scored under or equal to 50 on the global assessment of functioning scale (GAF). Other inclusion criteria were a minimum age of 16 years and sufficient understanding of the Dutch language. Five years later patients were asked to participate in the follow-up measure. Of the original sample of 323 patients, 78 did not participate in the follow-up measure because they could not be traced, were not willing to participate, had emigrated or were not able to do the interview because of severe symptomatology. Thirty patients had died between 2005 and the follow-up. Out of the remaining 215 participants, 124 patients were excluded because they did not meet the criteria of being an outpatient, but instead lived in psychiatric clinics or in a sheltered housing setting. This resulted in a total of 92 outpatients with SMI for this victimisation study. All SMI patients received Flexible Assertive Community Treatment (FACT) at Mentrum, the specialised department for SMI patients at Arkin Mental Health Care or at GGZinGeest [32,33]. FACT is a Dutch variation of Assertive Community Treatment (ACT). FACT combines two approaches within one multidisciplinary team: (1) individual case management for extensive care for SMI patients who are currently stable and (2) shared caseload with intensive full ACT approach for SMI patients with more mental health care needs. In this way FACT allows patients to stay in the team during more stable periods and therefore ensuring continuity of mental health care. FACT provides support on a wide variety of living areas and unrestricted access to care if needed [33].

#### Control group

Data of the psychiatric outpatients was compared with IVM data of the general population. In this study, we only use data of the 10865 respondents who live in the Amsterdam area [34]. IVM surveys were distributed via the internet or on paper depending on the participants’ preference. People who did not respond to the internet survey were phoned or visited at their homes.

### Measures

Past year victimisation was measured with Section 4 of the Dutch Integral Safety Monitor *(Integrale Veiligheidsmonitor*, IVM) developed by the Dutch Ministry for Security and Justice [34]. The IVM is a self-report safety survey that is annually conducted in the Netherlands by the Statistical Centre for Civilian Demographic Research (CBS). National safety surveys, such as the IVM, are currently the most comprehensive instruments to assess victimisation [11]. The IVM is used to assess feelings of safety in the Netherlands in almost 65000 households every year and is the most reliable victimisation measure that is currently available in the Netherlands [34]. In this study, we only use data of respondents who live in the Amsterdam area (n = 10,865). A strength of the IVM is that municipal councils can voluntarily participate in the IVM study, and via oversampling get data from groups with high non-response rates in the national survey. The IVM assesses victimisation on fourteen specific crimes, which can be classified into three main categories: violent crimes (sexual crimes, threats and assault), property crimes (burglary, theft and pickpocketing) and vandalism. For each specific crime participants were asked if they had been victimised in the five years preceding the interview. In case of a positive answer, patients were asked in which year and month the two most recent victimisation incidents took place. These questions were solely intended to reduce recall bias and were not used for analysis. Subsequently, it was asked if victimisation of that specific crime had happened in the last twelve months (12 month prevalence). Finally, for the violent crimes we asked where the victimisation had taken place and whether the perpetrator was a relative, an acquaintance or a stranger.

Personal and socio-demographical information was assessed with Section 12 of the IVM survey. Information regarding social, occupational and psychological functioning was obtained using the modified Global Assessment of Functioning scale [35]. Duration of the treatment and comorbid substance use was assessed via the electronic patient file of the mental healthcare setting. Psychopathology was measured with several items derived from the Expanded Brief Psychiatric Rating Scale (BPRS-E) [36]. Some BPRS items are typical for a specific diagnostic group that would distort the regression analysis. Therefore, we only use items with sufficient variance in each group. BPRS items that were scored 'not present' in 90% or more of the participants from a diagnostic group were not used in the regression analysis. Items used were: Hostility; Unusual thought content, Tension, Excitement, Distractibility and Motor hyperactivity.

### Medical ethics issues

The research is in compliance with the Helsinki Declaration, which contains ethical principles for medical research involving human subjects. The Medical Ethics Review Committee of VU University Medical Centre, a Dutch medical ethics committee, gave permission to conduct the surveys and decided that the Dutch Medical Research Involving Human Subjects Act (WMO) does not apply to this study and that official approval of the study by their committee is not required.

### Statistical analysis

Statistical analyses were conducted using SPSS Statistics for Microsoft Windows [37], a computer software package for statistical analysis. Between group analyses were conducted using the Pearson Chi-square test for dichotomous variables, and ANOVA for continuous variables, Effect sizes for dichotomous variables were obtained with Cramer’s V test. The Bonferroni Test was used as a *post hoc* test for continuous variables. For comparison of the patient group with the general population we also used a Pearson Chi-square test. To correct for differences between the two samples, IVM data of the general population was weighed for age, gender, ethnicity, level of education and living area.

Associations of socio-demographics, clinical variables and substance use were analysed for violent and non-violent crimes within the patient group. First, a univariate analysis was conducted. Furthermore, to examine associations between psychopathology and victimisation, univariate regression analyses were performed for the different BPRS items. Additional univariate regression analysis was performed to examine the association of co-morbid substance use with victimisation; this was done only for the Depression and SMI subgroup due to multicollinearity with the SUD subgroup. Subsequently, a multivariate hierarchical logistic regression analysis was conducted (method ENTER). In this analysis two separate groups of independent variables were added subsequently. In step 1, socio-demographic characteristics were entered (age, gender, ethnicity, education, living alone and employment); in step 2 the diagnostic subgroup was entered (SMI, Depression or SUD). In all analyses the level of significance used was p<0.05.

## Results

### Sample characteristics

Characteristics of included participants are presented in Table 1. Statistical analysis showed that the diagnostic subgroups differ in age, gender, ethnicity, education, living situation, employment, co-morbid substance use disorder, duration of treatment and BPRS items (p<0.05). Samples seem representative for the diagnostic subgroups in the Dutch population [38].

**Table 1:** Socio-demographics and substance use characteristics

	Depression	Substance use disorder	Severe mental illness	χ2/p^*^ (Effect size)
Characteristics	(n = 102)	(n = 106)	(n = 92)	
Age, mean (SD)	43.8 (9.2)	41.2 (9.4)	50.5 (8.7)	.000^b^ ^c^ (.155)^e^
Gender, n (%)				
Male	34 (33.3)	80 (75.5)	46 (50.0)	
Female	68 (66.7)	26 (24.5)	46 (50.0	000^a^ ^b^ ^c^ (.354)^d^
Ethnicity, n (%)				
Western	57 (55.9)	79 (74.5)	62 (67.4)	
Non-Western	45 (44.1)	27 (25.5)	30 (32.6)	017^a^ (.165)^d^
Primary / secondary education only, n (%)	41 (40.2)	57 (53.8)	66 (71.7)	.000^a^ ^b^ ^c^ (.255)^d^
Living alone, n (%)	38 (37.3)	59 (55.7)	71 (77.2)	.000^a^ ^b^ ^c^ (.323)^d^
Employed, n (%)	38 (37.3)	32 (30.2)	15 (16.3)	.013^b^ ^c^ (.190)^d^
Co-morbid substance use disorder, n (%)	6 (5.9)	-	23 (25.0)	.000^a^ (.266)^d^
Duration of treatment in years, mean (SD)	1.5 (2.3)	0.8 (1.6)	12.4 (5.5)	.000^b^ ^c^
Items BPRS-E, mean (SD)				
Hostility	2.9 (1.6)	2.1 (1.1)	1.5 (0.9)	.000^a^ ^b^ ^c^ (.195)
Unusual thought content	1.2 (0.5)	1.0 (0.2)	1.9 (1.5)	.000^b^ ^c^ (.143)
Blunted affect	1.2 (0.7)	1.4 (0.7)	1.5 (0.9)	.002^b^ (.028)
Tension	1.1 (0.2)	1.2 (0.5)	1.5 (1.0)	.000^b^ ^c^ (.060)
Excitement	1.1 (0.3)	1.3 (0.6)	1.3 (0.6)	.002^a^ ^b^ (.042)
Distractibility	1.1 (0.3)	1.1 (0.4)	1.3 (0.6)	.003^b^ ^c^ (.038)
Motor hyperactivity	1.1 (0.3)	1.6 (1.0)	1.4 (0.9)	.000^a^ ^b^ (.087)

* p is a result of ANOVA for BPRS items and χ^2^ test for categorical variables for differences between Depression, SUD & SMI.

^a^ Statistical analysis indicates a significant difference between the depression and the SUD group (asymptotic (2-sided) < 0.05).

^b^ Statistical analysis indicates a significant difference between the depression and the SMI group (asymptotic (2-sided) < 0.05).

^c^ Statistical analysis indicates a significant difference between the SUD and the SMI group (asymptotic (2-sided) < 0.05).

^d^ Cramer’s V

^e^ Eta squared (η^2^)

### Victimisation prevalence in psychiatric outpatients and the general population

As summarized in Table 2, we found that prevalence of overall victimisation in psychiatric outpatients living in the Amsterdam area is 1.3 times higher than the prevalence in the weighed sample of the general population of Amsterdam area (p<0.001). Sixty-one percent of the psychiatric outpatients reported at least one instance of victimisation in the last year, compared to 46.9% in the general population. Furthermore, the prevalence of violent victimisation was higher amongst psychiatric outpatients than the general public (p<0.001). Thirty-three percent of the patients reported to have fallen victim to at least one violent crime, compared to 9.5% in the control group, which is a risk ratio of 3.5. Property crimes were reported by 36.3% of the patients, compared to 29.2% in the control group (p = 0.02), which is a risk ratio of 1.2.

**Table 2:** Twelve month prevalence rates of victimisation of patients with depression, substance use disorder and severe mental illness, psychiatric patients overall and the general population.

	Depression	Substance use disorder	Severe mental illness	χ^2^	Overall patients	Weighed general population Amsterdam district ^d^	χ^2^	Risk Ratio^‡^
	(n = 102)	(n = 106)	(n = 92)	(Cramer’s V)	(n = 300)	(n = 10865)	(Phi)	
Type of crime	% (95% CI)	% (95% CI)	% (95% CI)		% (95% CI)	% (95% CI)		
All crimes	66.7 (57.4–76)	75.5 (67.1–83.8)	41.3 (31.1–51.6)	.000^b^ ^c^ (.293)	62.0 (56.5–67.5)	48.6 (47.6–49.5)	.000 (-.044)	1.3
Violent crimes	34.3 (24.9–43.7)	42.5 (32.9–52)	21.7 (13.2–30.3)	.008^b^ ^c^ (.179)	33.3 (28–38.7)	10.1 (9.5–10.6)	.000 (-.122)	3.3
Sexual offences	6.9 (1.9–11.9)	6.6 (1.8–11.4)	3.3 (-0.4–7)	.489 (.069)	5.7 (3–8.3)	2.2 (1.9–2.5)	.000 (-.038)	2.6
Threats	22.5 (14.3–30.8)	30.2 (21.3–39.1)	17.4 (9.5–25.3)	.069^c^ (.123)	23.7 (18.8–28.5)	7.5 (7–8)	.000 (-.097)	3.2
Assaults	10.8 (4.7–16.9)	17 (9.7–24.2)	7.6 (2.1–13.1)	.069^c^ (.120)	12.0 (8.3–15.7)	2.4 (2.1–2.7)	.000 (-.096)	4.9
Property crimes	33.3 (24–42.6)	55.7 (46–65.3)	17.4 (9.5–25.3)	.000^a^ ^b^ ^c^ (.325)	36.3 (30.9–41.8)	30.5 (29.6–31.3)	.030 (-.021)	1.2
Attempted Burglary	5.9 (1.2–10.5)	5.7 (1.2–10.1)	3.3 (-0.4–7)	.654 (.053)	5.0 (2.5–7.5)	5.6 (5.2–6)	.655 (.004)	ns
Burglary	3.9 (0.1–7.8)	3.8 (0.1–7.5)	0.0 (NA)^#^	.162 (.110)	2.7 (0.8–4.5)	3.0 (2.6–3.3)	.765 (.003)	ns
Bicycle theft	13.7 (6.9–20.5)	30.2 (21.3–39.1)	10.9 (4.4–17.4)	.001^a^ ^c^ (.221)	18.7 (14.2–23.1)	13.6 (13–14.3)	.013 (-.024)	1.4
Car theft and theft from car	2.9 (-0.4–6.3)	3.8 (0.1–7.5)	0.0 (NA)^#^	.189 (.105)	2.3 (0.6–4.1)	7.5 (7–8)	.001 (.032)	0.3
Pickpocketing	3.9 (0.1–7.8)	13.2 (6.7–19.8)	2.2 (-0.9–5.2)	.003^a^ ^c^ (.196)	6.7 (3.8–9.5)	5.1 (4.6–5.5)	.211 (-.012)	ns
Robbery	0.0 (NA)^#^	2.8 (-0.4–6)	1.1 (-1.1–3.2)	.199 (.104)	1.3 (0.0–2.6)	1.0 (0.8–1.1)	.507 (-.006)	ns
Other theft	10.8 (4.7–16.9)	13.2 (6.7–19.8)	2.2 (-0.9–5.2)	.019^b^ ^c^ (.163)	9.0 (5.7–12.3)	6.6 (6.1–7)	.097 (-.016)	ns

^a^ Pearson Chi-square indicates a significant difference between the depressive and the SUD group (asymptotic (2-sided) < 0.05).

^b^ Pearson Chi-square indicates a significant difference between the depression and the SMI group (asymptotic (2-sided) < 0.05).

^c^ Pearson Chi-square indicates a significant difference between the SUD and the SMI group (asymptotic (2-sided) < 0.05).

^d^ IVM data was weighed for gender, age, ethnicity, level of education and living area.

‡ Ratio of overall reported prevalence in psychiatric patients to prevalence reported by general population

# The sample rate is 0; confidence bounds are not reported.

### Victimisation prevalence in different diagnostic key-groups

Differences between the diagnostic groups were found for violent victimisation, as well as for victimisation of property crimes (Pearson Chi-square, p<0.01) (Table 2). *Post hoc* analysis revealed that patients with depression and patients with SUD reported 1.6 to 1.8 times more overall victimisation than SMI patients (p<0.001) and 1.6 to 2.0 times more violent victimisation than the SMI group (p>0.05). With regard to property crimes we found that victimisation was the highest in the SUD group (55.7%). Patients with SUD reported 3.2 times more property victimisation than the SMI group and 1.7 times more than depressive patients.

### Contextual aspects of violent victimisation

Analysis of the contextual aspects of the violent victimisation revealed that the majority of incidents took place in public spaces (49.2%) (Table 3). The perpetrators were predominantly strangers to the patients (41.1%). Differences between the diagnostic subgroups were found with regard to the location of the last reported violent victimisation (p = 0.014). Patients with SUD reported that 59.3% of violent victimisation occurred in public spaces. The patients with depression also reported public places as most common location of crime (47.5%), but also reported their own home in 30.0% of the violent crimes. In the group of patients with SMI their own home (54.5%) was the most reported location, followed by public space (31.8%). With regard to the perpetrator of the last reported violent victimisation no differences were found (Table 3). In all groups, violent crimes were mainly committed by strangers, partners or ex-partners.

**Table 3:** Location and perpetrator of most recent violent victimisation incident (sexual offences, threats and assaults) for patients with depression, substance use disorder and severe mental illness.

	Depression	Substance use disorder	Severe mental illness	χ^2^ (Cramer’s V)
Location (%)				
Public space^‡^	47.5	59.3	31.8	
Own home	30.0	11.9	54.5	
Other home	0.0	10.2	4.5	
Bar, restaurant or shop	10.0	5.1	4.5	
Work or school	10.0	8.5	0.0	
Elsewhere	2.5	5.1	4.5	0.014 (.303)
Perpetrator (%)*				
Stranger	52.5	35.6	40.9	
Partner or ex-partner	25.0	10.2	13.6	
Neighbour	15.0	23.7	27.3	
Relative	0.0	3.4	4.5	
Other acquaintance	7.5	27.1	13.6	0.104 (.234)

^‡^ Public space includes: victimisation in streets, public transport, parks, parking lots and beaches.

* 3 missing values

### Univariate and multivariate analyses

Table 4 shows the results of univariate analysis off the association between different patient characteristics and victimisation. Univariate analysis revealed that being of younger age (p = 0.001, Exp B = 0.958) and having a diagnosis of SUD (p = 0.002, Exp B = 2.656) are associated with violent victimisation. Being male (p = 0.005, Exp B = 0.496), not living alone (p = 0.004, Exp B = 0.494), having a diagnosis of depression (p = 0.012, Exp B = 2.375) and a diagnosis of SUD (p<0.001, Exp B = 5.963) are associated with victimisation related to property crimes. We performed a univariate analysis for the association of co-morbid substance use for patients with depression and SMI. We found no association with violent victimisation or with property victimisation. Furthermore, we found that BPRS item Hostility as significant predictor for violent victimisation (p<0.001, Exp B = 1.40). For the remaining BPRS items no significant results were found. With regard to property crimes, Unusual Thought Content (p = 0.014, Exp = 0.62) and Distractibility (p = 0.033, Exp B = 0.50) were found to be significantly associated with higher victimisation.

**Table 4:** Results of univariate and multivariate hierarchical logistic regression analyses (method ENTER) for predictors of violent victimisation and victimisation of property crimes in psychiatric patients (n = 300).

	Univariate		Multivariate			
			Step1		Step 2	
Variables (reference category)	Exp B	p	Exp B	p	Exp B	P
Violent crimes						
Age	0.958	**.001**	0.960	**.003**	0.968	**.025**
Gender (female)	0.754	.252	0.760	.285	0.829	.502
Ethnicity (non-white western)	1.220	.438	1.179	.542	1.229	.455
Education (more than secondary)	1.408	.164	1.245	.389	1.192	.503
Living alone	0.833	.459	0.954	.857	1.027	.921
Employed	1.325	.294	1.134	.653	1.120	.691
Diagnosis						
SMI	REF	REF	-		REF	REF
Depression	1.881	.054	-		1.434	.334
SUD	2.656	**.002**	-		1.798	.106
Property crimes						
Age	0.977	.064	0.985	.265	1.010	.518
Gender (female)	0.496	.005	0.459	.002	0.612	.085
Ethnicity (non-white western)	0.996	.988	0.866	.594	1.033	.909
Education (more than secondary)	1.231	.387	1.141	.602	1.086	.760
Living Alone	0.494	.004	0.462	.003	0.508	.015
Employed	1.236	.423	0.993	.979	0.975	.932
Diagnosis						
SMI	REF	REF	-		REF	REF
Depression	2.375	**.012**	-		2.089	.060
SUD	5.963	**.000**	-		5.292	**.000**

Step 1 Violent crimes: Omnibus test; Step P = 0.025, Model P = 0.025. Hosmer en Lemeshow; P = 0.901. Nagelkerke; R2 = 0.066.

Step 2 Violent crimes: Omnibus test; Step P = 0.265, Model P = 0.029. Hosmer en Lemeshow; P = 0.860. Nagelkerke; R2 = 0.078.

Step 1 Property crimes: Omnibus test; Step P = 0.002, Model P = 0.002. Hosmer en Lemeshow; P = 0.995. Nagelkerke; R2 = 0.090.

Step 2 Property crimes: Omnibus test; Step P = 0.014, Model P = 0.000. Hosmer en Lemeshow; P = 0.324. Nagelkerke; R2 = 0.181.

A multivariate hierarchical logistic regression analysis (method ENTER) was carried out for violent victimisation and victimisation of property crimes for the variables that were also used for univariate analysis (Table 4). In the first step a model was calculated for different socio-demographic variables. Subsequently, in step 2 we added the diagnosis. The first model is used to analyse possible predictors for violent victimisation. Being of young age was found to be a significant predictor (p = 0.003, B = 0.960) of violent victimisation in the first step. Other patient characteristics were not significant predictors of violent victimisation. Step 2 revealed a final multivariate model for violent victimisation in which being of young age (p = 0.025, B = 0.968) remained as the only significant independent predictor. With regard to property crimes, being male (p = 0.002, B = 0.459) and not living alone (p = 0.003, B = 0.462) remained as significant independent predictors of victimisation in the first step of the analysis. In the second step we found not living alone (p = 0.015, B = 0.508) and having a diagnosis of SUD (p<0.001, B = 5.292) as significant independent predictors.

## Discussion

### Main findings

Our results show that psychiatric outpatients are at increased risk of falling victim to both violent and non-violent crimes. Prevalence rates for violent victimisation were 3.5 times higher in psychiatric outpatients than in the general population. A total of 33% of psychiatric outpatients reported to have been violently victimised, which includes sexual crimes (6%), threatened assault (24%) and physical assault (12%). Furthermore, 36% of the psychiatric outpatients reported at least one property crime, which is 1.2 times higher than the general population. This increased prevalence of victimisation cannot be explained by differences in age, gender, ethnicity, level of education, living area or education level since the data of the general population was weighed for these variables. This is the first study to assess victimisation in psychiatric outpatients with depression and substance use disorder in The Netherlands. Our findings are in line with other victimisation prevalence studies in psychiatric outpatients conducted in Europe [5,7,8,11], the United States [1,14,39] and Australia [12,18].

### Victimisation prevalence in different diagnostic groups

We found differences in victimisation between the diagnostic subgroups. Prevalence rates in outpatients with depression or SUD were higher on overall victimisation (respectively 67% and 76%) than in outpatients with SMI (41%). In our study SMI was defined as a DSM-IV diagnosis of schizophrenia, schizoaffective disorder or bipolar disorder [30] and continuous intensive mental health care for more than two years. Moreover, all included patients had to live independently in the community. Besides psychopathological differences, some aspects of treatment may contribute to these differences. SMI patients received FACT treatment, a Dutch variation of ACT. FACT is, in comparison with the treatments used for the depressive and SUD patients, a more extensive form of treatment. It provides support on a wide variety of living areas and unrestricted access to care if needed. FACT results in better therapy adherence and higher levels of social-functioning compared to regular ambulant therapy [33]. Therefore it is likely that FACT plays a role in the low victimisation prevalence in the SMI group. However, scientific research has not jet addressed this issue. Furthermore, the differences in treatment duration can have affected the prevalence of victimisation. Patients with a depression or SUD may not have received treatment during the entire 12 months which were assessed in this study, while the SMI patients did.

More than a third of the depressive outpatients reported to have fallen victim to a violent crime in the past year. These results are striking since there seems to be little attention for victimisation in depressed outpatients in clinical settings. Current victimisation research seems to focus only on patients with SMI, SUD, or a combination of those two. Existing knowledge concerning victimisation in patients with depression is minimal. Our findings show that victimisation in depressed patients is a serious problem. Two previous studies have addressed this topic [24,25]; both report increased victimisation in depressed patients, consistent with our findings. As a result of a gap in scientific literature regarding the underlying mechanisms of victimisation in this group, we can only speculate on the explanation. The increased victimisation rates may be explained by the stress generation hypothesis [40,41]. According to this hypothesis, individuals with depression experience higher rates of negative and stressful life events triggered by their own behaviour. Depressed patients are more often agitated, cynical and irritable. As a result, interpersonal relationships are more likely to be aversive to others, which may lead to anger, anxiety and feelings of sadness [42]. This can in turn provoke conflict situations that result in victimisation. Moreover, depressed patients are likely victimised more often because they do not avoid threatening situations as a result of negative cognitive schemas, such as feelings of hopelessness and guilt [43,44]. Apart from depression leading to increased victimisation, victimisation may in turn increase the chance of having a relapse or continuation of depressive symptoms, resulting in a vicious circle of depression-stress-depression. Our finding that depressed patients are often victimised at home by a partner or ex-partner supports this hypothesis. Another possible explanation is that the high rates of victimisation in this group are caused by cognitive deficits in executive functioning, memory and attention that are associated with depression [45]. These deficits may result in a reduced ability to recognize dangerous situations and decreased competence to cope with them [46]. It is important that such hypotheses receive more attention in future studies, since more knowledge about the underlying mechanisms is essential for the development of successful prevention programs.

We also found higher prevalence of overall victimisation in patients with SUD (76%) than in those with SMI (41%). Violent victimisation was more than twice as prevalent in the SUD group (43%) as in the SMI group (22%). Moreover, 56% of the patients with SUD reported victimisation of property crimes in the last year, compared to 33% in the depressed patients and 17% in the SMI patients. Other studies have also found that substance abuse is associated with an increased risk of victimisation [1,7,9,18,19].

Stevens et al. [23] offer three possible explanations for the high prevalence of victimisation in patients with SUD. The first explanation assumes that the drug use itself causes increased risk of victimisation. In this scenario, the disinhibiting effects of alcohol and drugs cause judgment errors in interpersonal contact, and reduce the patient’s ability to recognize threatening situations. This explanation fits with the *route activity theory* [47], which states that people with SUD are prone to victimisation because they spend much time in groups that include many perpetrators and few ‘capable guardians’. We found that SUD patients are most often victimised in public spaces, by strangers and acquaintances such as people they know from the streets. Thus, our results appear to support this theory. The second explanation suggests that the use of addictive substances is a consequence of victimisation. Exposure to physical and sexual violence is associated with feelings of sadness, humiliation, and powerlessness [23,27,28]. As a consequence, victims may try to compensate these negative emotions through the use of psychoactive substances. Since our study is based upon a cross-sectional design it is not possible to draw causal conclusions that either support or contradict this explanation. However, we find that a big proportion of patients that are already in treatment for SUD report victimisation in the past twelve months, thus experiencing victimisation after the onset of their SUD. Therefore it seems unlikely that this explanation can account for all victimisation in this group. Finally, the third, and according to Stevens et al. [23] the most likely explanation, is that both the victimisation and the SUD are caused by confounding factors, such as poor mental health [23], social exclusion [48], employment problems, interpersonal difficulties, and childhood family violence [49]. However, we found that both social exclusion and employment problems were more frequent in the group of SMI patients than in the SUD patients. Since the SMI patients reported less victimisation than the SUD patients, it seems likely that there are other factors that cause the high prevalence rates in the SUD group. We suspect that the confounding factors could cause a vicious cycle in which victimisation and use of addictive substances reinforce each other, probably by both ways described above.

Although prevalence of violent victimisation is lower in SMI patients than in patients with SUD and depressed patients, it is still high compared to the general population (22% vs. 10%). The relatively low prevalence of victimisation in the SMI group may be related to the fact that we only included outpatients, who were living independently in the community. The body of research that addresses differences in victimisation between in- and outpatients is small and results of studies that use only inpatients versus those who focus on outpatients are hard to compare due to methodological differences. We do know however that symptom severity is related to victimisation [1,9], and inpatients are therefore more likely to be victimised. Victimisation among inpatients may also be affected by other factors such as staff supervision and closeness to other patients. It is therefore important to note that our results only reflect victimisation rates for outpatients. Surprisingly, SMI patients report less property crimes than the general population, probably due to the fact that most SMI patients do not own much expensive property, such as cars. Since we do not have access to data on property ownership it was not possible to control for this. It is not clear what mechanisms put patients with SMI at increased risk of victimisation. In a systematic review on victimisation in SMI patients, Maniglio [21] offers two hypotheses for the increased risk. First he states that severe psychiatric symptoms which are common in SMI patients, such as poor reality testing, disorganisation, impulsivity and paranoia cause judgment errors in social situations resulting in a reduced ability to recognize and avoid threatening situations. Our findings support this hypothesis, since we find that severity of symptoms is associated with victimisation. Secondly, he states that SMI patients are at increased risk because they encounter more dangerous places and situations, because of their homelessness and use of intoxicating substances. However, our results contradict this hypothesis, since most SMI patients are victimised in their own home by strangers and neighbours. We believe that another explanation can be found in interaction with others. SMI patients can exhibit bizarre, antisocial, aggressive or provocative behaviour [50]. Their typical, sometimes inappropriate and awkward behaviour is often misinterpreted by others and unintentionally provokes aggressive behaviour [51], thereby increasing the likelihood of interpersonal conflict [51]. Our finding that SMI patients are often victimised by strangers supports this hypothesis.

### Factors associated with victimisation

We found that a diagnosis of SUD was associated with violent victimisation. This result accords with previous studies [1,7,9,18,19], but it disappeared in our final multivariate model once socio-demographic characteristics have been taken into account. We also did not found an association between co-morbid substance use and violent victimisation in patients with depression or SMI. The finding that younger psychiatric patients have an increased risk for violent victimisation, matches findings from Dean and colleagues [5], who identified young age as one of the predictors for violent victimisation. Furthermore, we found that patients who exhibit hostile behaviour have an increased risk of being violently victimised. These findings are in line with earlier studies that have identified severity of psychiatric symptoms as a predictive factor [1,9,20]. However, we did not find differences in victimisation related to the other measured symptoms, such as unusual thought content, blunted affect, tension, motor hyperactivity and excited behaviour. Possibly this can be explained by our selection solely of outpatients, resulting in a smaller range of severity of psychiatric symptoms compared to the general population of psychiatric patients. It is plausible that this selection makes it hard to find any statistically significant correlations between severity of symptoms and victimisation. This hypothesis agrees results from another study amongst outpatients that did not find a significant relationship between specific psychiatric symptoms and victimisation [52], whilst studies amongst inpatients or homeless patients do find associations between specific symptoms and victimisation [53–55]. Ethnicity was not found to be a predictive factor, in accord with earlier findings [5]. We did not identify gender as a predictor for overall victimisation. While in the general population men are consistently found to be at elevated risk to overall victimisation, compared to women, this pattern is not found in psychiatric patients [56]. Previous literature reports inconsistent findings on sex differences in victimisation prevalence in psychiatric patients. Six previous studies found no differences between men and women for overall violent victimisation [4,5,9,14,19,55]. Three studies found a higher prevalence in men [7,9,29] and one study found higher prevalence in women [10]. Although Teplin et al. [14] did not find gender differences in overall violent victimisation, they found that men had experienced more violent robbery and women had experienced a higher risk of being sexually assaulted.

With regard to non-violent victimisation it is difficult to make comparisons with earlier studies because violent and non-violent victimisation are often grouped as a single outcome. Furthermore, most studies in the field of victimisation of psychiatric patients have focused on violent victimisation. Thus, little is known about risk factors in patients for property crimes. We found that male patients had a higher risk of falling victim to property crimes compared to women. This finding is in line with Stevens et al. [23], who found that being male is associated with experiencing property crimes in patients with SUD. Teplin et al. [14] found that women reported more theft of motor vehicles than men. However, when we added the diagnosis of the patients to the model in the multivariate analyses, the predictive value of gender was no longer significant. Furthermore, we found that not living alone and having a diagnosis of SUD can be seen as risk factors for property crimes. We did not control for the amount of property owned by the patients. It is likely that because of their lower financial income, patients with psychiatric disorders, and especially SMI patients, possess less property. This is likely to influence victimisation rates for property crimes.

### Limitations

This study has some limitations that need further consideration. First, because this study is cross-sectional it is not possible to make causal interpretations about victimisation and psychopathology. Future studies with prospective and longitudinal designs may provide more understanding of underlying mechanisms. Such knowledge can contribute to the development of targeted victimisation prevention programs. Second, this study is entirely based on self-reported data. Although self-report measures, particular national crime surveys, are seen as the golden standard of measuring victimisation in patients [11,57], it is possible that results were influenced by memory bias of participants. Still, Teplin et al. [14] found that recall bias (e.g. not remembering victimisation incidents within the time frame) was greater than the bias of telescoping, such as recalling victimisation incidents that occurred prior to the time frame. Thus, if reports of patients are biased, our results may be an underestimation of victimisation prevalence rates indicating that the problem of victimisation is even bigger. Third, our control group consisted of a random sample of respondents from the general population in Amsterdam. Information about psychiatric disorders or substance use for the general population was not available. It is therefore important to note that we were not able to compare psychiatric patients with respondents without any psychiatric or substance use problems. Finally, only patients that were willing to cooperate participated in the study. Therefore, it is possible that patients with problems such as extreme apathy and severe paranoia were not included. Since severity of symptoms is likely to be a risk factor for victimisation [1,9,20], this again would indicate that our results are conservative. Despite these limitations, our study is the first to compare violent and non-violent victimisation of different diagnostic subgroups to the general population. The results indicate that prevalence of victimisation is high not only in outpatients with SMI or SUD, but also in outpatients with depression. Both for violent and non-violent crimes psychiatric outpatients are at increased risk of victimisation. Improving our understanding of differences in victimisation between diagnostic subgroups can contribute to the development of targeted prevention programs and increased awareness among patients, relatives, health care professionals and police.
